# Pathogenic missense mutation pattern of forkhead box genes in neurodevelopmental disorders

**DOI:** 10.1002/mgg3.789

**Published:** 2019-06-14

**Authors:** Lin Han, Meilin Chen, Yazhe Wang, Huidan Wu, Yingting Quan, Ting Bai, Kuokuo Li, Guiqin Duan, Yan Gao, Zhengmao Hu, Kun Xia, Hui Guo

**Affiliations:** ^1^ Center for Medical Genetics & Hunan Provincial Key Laboratory of Medical Genetics, School of Life Sciences Central South University Changsha China; ^2^ Center of Children Psychology and Behavior The Third Affiliated Hospital of Zhengzhou University Zhengzhou China; ^3^ Child Psychobehavioural Rehabilitation Department Shenzhen Baoan Maternal and Child Health Hospital Shenzhen China; ^4^ Key Laboratory of Medical Information Research Central South University Changsha China; ^5^ CAS Center for Excellence in Brain Science and Intelligences Technology (CEBSIT) Shanghai China

**Keywords:** de novo, forkhead box domain, *FOXG1*, *FOXP1*, *FOXP2*, missense variant, neurodevelopmental disorders

## Abstract

**Background:**

Forkhead box (FOX) proteins are a family of transcription factors. Mutations of three FOX genes, including *FOXP1*, *FOXP2*, and *FOXG1,* have been reported in neurodevelopmental disorders (NDDs). However, due to the lack of site‐specific statistical significance, the pathogenicity of missense mutations of these genes is difficult to determine.

**Methods:**

DNA and RNA were extracted from peripheral blood lymphocytes. The mutation was detected by single‐molecule molecular inversion probe‐based targeted sequencing, and the variant was validated by Sanger sequencing. Real‐time quantitative PCR and western blot were performed to assay the expression of the mRNA and protein. To assess the pattern of disorder‐related missense mutations of NDD‐related FOX genes, we manually curated de novo and inherited missense or inframeshift variants within *FOXP1*, *FOXP2*, and *FOXG1* that co‐segregated with phenotypes in NDDs. All variants were annotated by ANNOVAR.

**Results:**

We detected a novel de novo missense mutation (NM_001244815: c.G1444A, p.E482K) of *FOXP1* in a patient with intellectual disability and severe speech delay. Real‐time PCR and western blot revealed a dramatic reduction of mRNA and protein expression in patient‐derived lymphocytes, indicating a loss‐of‐function mechanism. We observed that the majority of the de novo or transmitted missense variants were located in the FOX domains, and 95% were classified as pathogenic mutations. However, 10 variants were located outside of the FOX domain and were classified as likely pathogenic or variants of uncertain significance.

**Conclusion:**

Our study shows the pathogenicity of missense and inframeshift variants of NDD‐related FOX genes, which is important for clinical diagnosis and genetic counseling. Functional analysis is needed to determine the pathogenicity of the variants with uncertain clinical significance.

## 1 INTRODUCTION

In 1990, Weigel and his colleagues identified a DNA binding domain (DBD) that was similar to HNF‐3 transcription factors (Weigel & Jackle, 1990). Thus, this domain was defined as a novel transcription factor family called forkhead box (FOX) proteins. FOX proteins have an evolutionarily conserved DNA binding domain called “forkhead” or “winged‐helix” that is involved in chromatin remodeling and nuclear localization. FOX proteins display a significant functional diversity and involved in diverse biological processes (Carlsson & Mahlapuu, 2002; Lam, Brosens, Gomes, & Koo, 2013). FOX genes have been associated with various diseases. Three FOX genes, including *FOXP1* (MIM:605,515), *FOXP2* (MIM:605,317), and *FOXG1* (MIM:164,874)*,* have been reported to be associated with neurodevelopmental disorders (NDDs). *FOXP1* and *FOXP2* cooperate in the regulation of non‐neural developmental processes (Shu et al., 2007). Mutations in *FOXP1* and *FOXP2* cause autism spectrum disorder (ASD) and language impairment (Girirajan et al., 2013; Hamdan et al., 2010; Horn et al., 2010; Lam et al., 2013; Lehmann, Sowden, Carlsson, Jordan, & Bhattacharya, 2003; S. J. Turner et al., 2013) and intellectual disability (ID), while mutations in *FOXG1* cause ASD, Rett syndrome, and West syndrome(Ariani et al., 2008; Bahi‐Buisson et al., 2010; Kortum et al., 2011; Mitter et al., 2018; Striano et al., 2011).

It is reported that hundreds of genes are associated with NDDs with the development of a well‐defined clinical cohort and widespread application and use of next‐generation sequencing. Meanwhile, many de novo mutations were identified within NDD genes. Due to a lack of site‐specific statistical significance, the pathogenicity of many variants, especially de novo missense and inframeshift variants, remains to be determined. This situation significantly challenges clinical diagnosis practice and genetic counseling. Here, by gene‐panel sequencing, we detected a novel de novo missense variant within *FOXP1* in a patient with ID and speech delay. With this initial finding, we systematically curated all reported disorder‐related missense variants in three NDD‐related FOX genes (*FOXP1*, *FOXP2*, *FOXG1*) from the literature. We subsequently investigated the distribution pattern of a missense variants and assigned the pathogenicity to each missense variant.

## 2 MATERIALS AND METHODS

### 2.1 Editorial policies and ethical considerations

This study was approved by the Human Ethics Committee of Center for Medical Genetics, Central South University. Written informed consent was obtained from the family.

### 2.2 Mutation detection and validation

Peripheral blood was collected from the proband and parents with written informed consent. DNA was extracted from the lymphocytes using a standard proteinase K digestion and phenol–chloroform method. The de novo missense mutation of *FOXP1* was detected by single‐molecule molecular inversion probe (smMIPs)‐based targeted sequencing, which has been described elsewhere. In summary, smMIPs were designed using MIPgen with an updated scoring algorithm. After amplification, libraries were sequenced using the Illumina HiSeq2500 platform. Incorrect read pairs and low‐quality reads were removed. Sequences were aligned against GRCh37 using BWA‐MEM (v.0.7.13) (Li & Durbin, 2010). Variants were called with FreeBayes (v.0.9.14) (Erik Garrison, 2012; Sanders et al., 2004). Variants with sequence coverage over tenfold and read quality over 20 were annotated with ANNOVAR (Wang, Li, & Hakonarson, 2010). Variants were validated by Sanger sequencing in both the proband and parents. Microsatellite analysis was applied to eliminate the potential nonpaternity of the variant in the family. Microsatellite loci were amplified by PCR using fluorescently labeled primers. The labeled products were analyzed by capillary electrophoresis using GeneMarker and the ABI 3730XL DNA Analyzer.

### 2.3 Real‐time PCR

Lymphoblastic cells were lysed in TRI Reagent Solution (Invitrogen 00623971). Total RNA was extracted according to the manufacturer's protocol. RNA was reverse‐transcribed into cDNA with Revert Aid First Strand cDNA Synthesis Kit (Thermo 00590615). Quantitative real‐time PCR was run in triplicate using a Roche LightCycler 96 and FastStart Essential DNA Green Master (Roche 06924204001). Data were normalized to β‐actin expression using the Δ*C_t_* method.

### 2.4 Western blot

Whole‐cell lysates were extracted by 2× SDS sample buffer (0.125 M Tris HCl, pH 6.8, 10% β‐mercaptoethanol, 4% SDS, 20% glycerol, 0.004% bromophenol blue) containing a protease inhibitor cocktail (Calbiochem 539131). Proteins were resolved by 8% SDS‐PAGE (Beyotime P0012AC) and transferred onto polyvinylidene fluoride. The membranes were reacted with FOXP1 antibody (Cell Signaling 2005). We used actin (Servicebio GB12001) as an internal control to normalize band intensity. The signals were visualized by using SuperSignal West Femto Maximum Sensitivity Substrate (Thermo 34095).

### 2.5 Annotation of missense variants and inframeshift variants of FOX genes in the literature

To identify and annotate the published missense and inframeshift variants within the three NDD‐related FOX genes (*FOXP1*, NM_001244814.1; *FOXP2*, NM_014491.3; *FOXG1*, NM_005249.4), we analyzed the de novo variants curated in a database that integrates genome‐wide sequencing studies with large‐scale cohorts, especially for NDDs (T. N. Turner et al., 2017). In addition, we curated the publications to collect sporadic reported cases. We only considered de novo missense variants or inherited missense variants that co‐segregate with phenotypes within families. All variants were re‐annotated by ANNOVAR (Wang et al., 2010). Pathogenicity assignment was performed following the American College of Medical Genetics and Genomics (ACMG) standards and guidelines (Richards et al., 2015).

## 3 RESULTS

### 3.1 Identification of a de novo pathogenic missense variant within *FOXP1*

**Figure 1:**
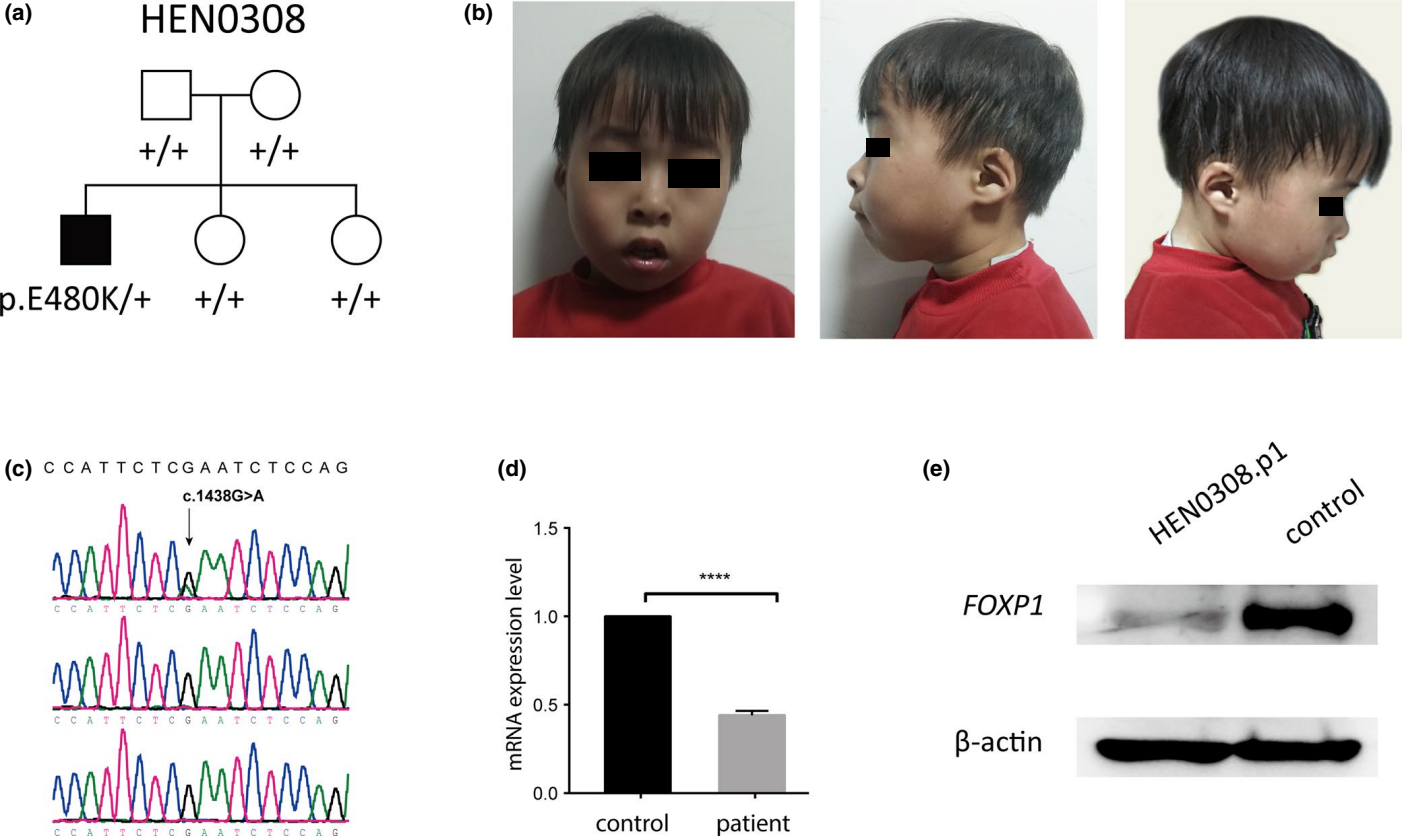
Identification of a novel de novo* FOXP1* missense variant in an intellectual disability patient. (a) Pedigree of the family. A heterozygous missense was detected in the proband but not parents. (b) Face view of the proband at 5 years of age. (c) Sanger validation of the missense variant in the proband and parents. Arrow indicates the site of the variant. (d,e) quantitative PCR and western blot assay detected a dramatic reduction in *FOXP1* mRNA and protein expression in the proband compared with the controls

The patient was a 5‐year‐old Han Chinese boy, 46,XY (Figure 1a). He was born via planned C‐section at 38 weeks. The patient weighed 3.0 kg and was 50 cm long at birth. At 2.5 years old, he had a height of 90 cm, a weight of 12 kg and an Head Circumference Z‐Score (HCZ) of 48.5 cm (−0.5 *SD*). The patient was diagnosed with ID and severe language delay. He raised his head at 1 month and turned over without the help of others at 3 month. He could grasp objects at 3 months. He was able to sit at 7 months and crawl and stand at 10 months. He began walking independently at 12 months and running and jumping at 2 years of age. However, he showed significant language development delay and experienced language regression at 2.4 years of age. He could only say simple words at the age of 3 years. Computed tomography showed normal results at 1.3 years of age. He had normal hearing, visual and vocal organs. When he was 5 years and 4 months old, his height was 116 cm, his weight was 19 kg and HCZ was 52 cm (+0.6 *SD*). The Wechsler Child Intelligence Scale showed his total IQ was 35, verbal IQ was <40, performance IQ was 44. The CRRC(S‐S) Language Development Check Scale showed his language level was <3 years old. His EEG and MRI results were normal. His hearing, visual and vocal organs were normal. He had a broad, prominent forehead, upturned nose, micromandible, and auricle valgus (Figure 1b). No other special facial deformities were observed. He had two healthy older sisters.

**Figure 2:**
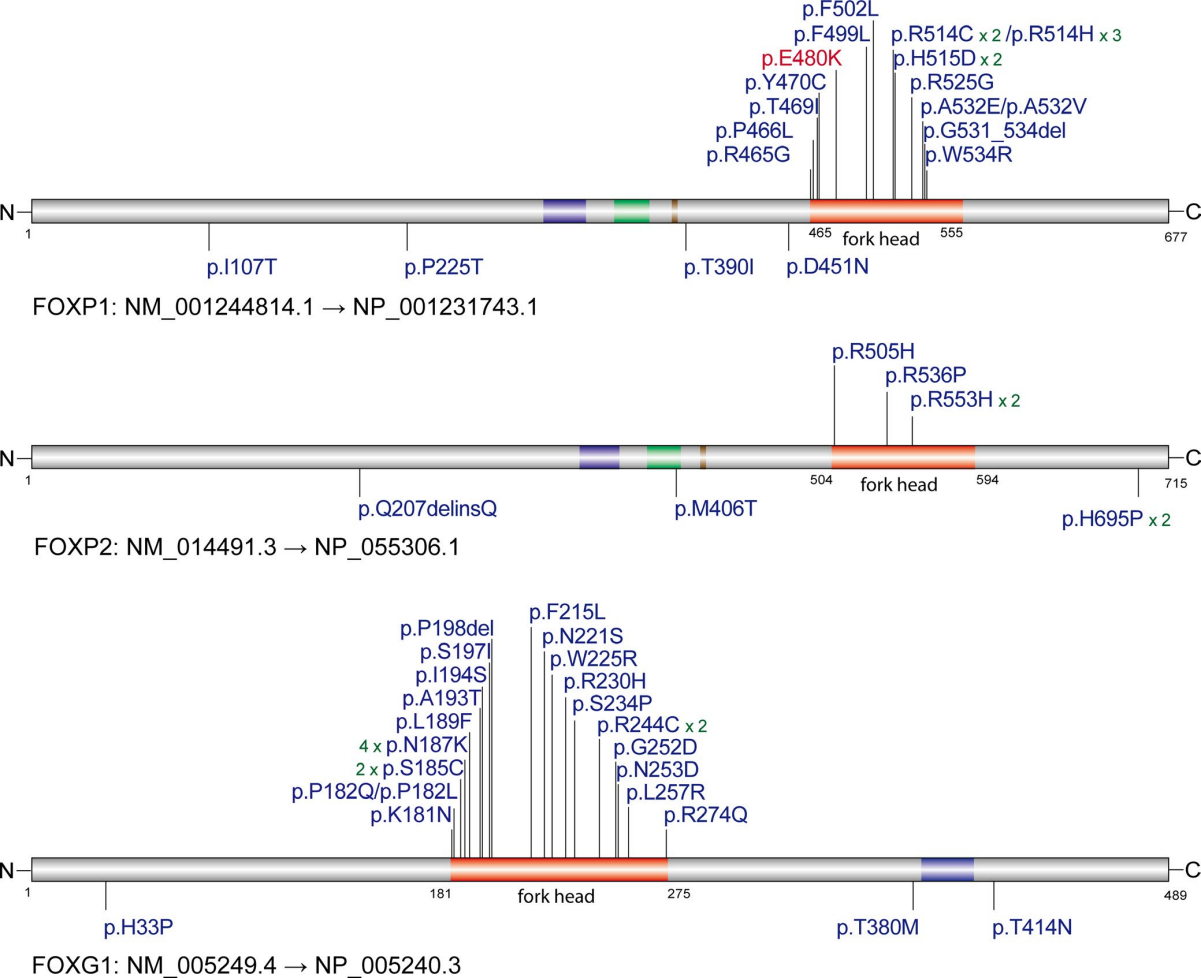
Protein diagram and variant locations of *FOXP1*, *FOXP2* and *FOXG1*. The variant with red color in *FOXP1* is from this study. Other variants were curated from the literature. Recurrent sites were indicated by numbers following the variant annotation (e.g., ×2 means this variant was detected in two independent patients). Significant missense clusters in the forkhead domains were observed in all three genes

Using smMIP‐based targeted sequencing, we detected a missense variant in *FOXP1* (NM_001244815, c.G1444A, p.E482K) (Figure 1c). This variant was absent in the parents and was confirmed as de novo. Nonpaternity was excluded. The variant is located in the forkhead domain of *FOXP1* (Figure 2). With the hypothesis that the missense variant causes an unstable mRNA, we performed a real‐time quantitative PCR (qPCR) assay on the mRNA extracted from the lymphocytes of the proband. qPCR analysis revealed that the mRNA in the proband was dramatically decreased (Figure 1d). We then performed a western blot to detect the protein expression in the patient's peripheral blood lymphocytes, which revealed a significant decrease in the patient compared with the control (Figure 1e), indicating a loss‐of‐function mechanism, which is consistent with the disruptive mutations.

### 3.2 Pathogenic missense/inframeshift variant pattern of NDD‐related FOX genes

In total, we curated 49 de novo missense/inframeshift variants in *FOXP1* (*n* = 19),* FOXP2* (*n* = 6) and *FOXG1* (*n* = 24) and four inherited missense variants co‐segregating with phenotypes in multigeneration families in *FOXP2* (Table 1). Ninety‐four percent (46/49) variants were absent in the Genome Aggregation Database (gnomAD). Most variants are predicted to be damaging by multiple in silico predictive programs (e.g., PolyPhen2, SIFT, and Mutation Taster) (Tables 1 and S1). After applying the ACMG guidelines, we classified 38/49 variants as pathogenic. In addition, 9/49 variants were classified as likely pathogenic, and 2/49 were classified as variants of uncertain significance (VUS) (Table 1).

**Table 1:** Missense variants of FOXP1, FOXP2, and FOXG1 and assigned clinical significance

NTchange	AAchange	Function	Inheritance	NumHit	PolyPhen2	SIFT	CADD1.3	Domain	gnomAD	Clinical significance
FOXP1										
c.320T>C	p.I107T	MIS	de novo	1	D	D	23.8	—	0	Likely pathogenic
c.673C>A	p.P225T	MIS	de novo	1	D	D	24.7	—	0	Likely pathogenic
c.1169C>T	p.T390I	MIS	de novo	1	P	D	23.4	—	0	Likely pathogenic
c.1351G>A	p.D451N	MIS	de novo	1	D	T	28.8	—	0	Likely pathogenic
c.1393A>G	p.R465G	MIS	de novo	1	D	D	26	Forkhead	0	Pathogenic
c.1397C>T	p.P466L	MIS	de novo	1	D	D	28.2	Forkhead	0	Pathogenic
c.1406C>T	p.T469I	MIS	de novo	1	D	D	27.2	Forkhead	0	Pathogenic
c.1409A>G	p.Y470C	MIS	de novo	1	D	D	26.2	Forkhead	1	Likely pathogenic
c.1438G>A	p.E480K	MIS	de novo	1	D	D	34	Forkhead	0	Pathogenic
c.1497T>A	p.F499L	MIS	de novo	1	D	D	25.2	Forkhead	0	Pathogenic
c.1506C>G	p.F502L	MIS	de novo	1	D	D	25.4	Forkhead	0	Pathogenic
c.1541G>A	p.R514H	MIS	de novo	3	D	D	34	Forkhead	0	Pathogenic
c.1540C>T	p.R514C	MIS	de novo	2	D	D	34	Forkhead	0	Pathogenic
c.1543C>G	p.H515D	MIS	de novo	2	D	D	27.7	Forkhead	0	Pathogenic
c.1574G>A	p.R525Q	MIS	de novo	1	D	D	34	Forkhead	0	Pathogenic
c.1595C>A	p.A532E	MIS	de novo	1	D	D	28.8	Forkhead	0	Pathogenic
c.1595C>>T	p.A532V	MIS	de novo	1	D	D	28.8	Forkhead	0	Pathogenic
c.1600T>C	p.W534R	MIS	de novo	1	D	D	26.8	Forkhead	0	Pathogenic
c.1590_1601del	p.G531_534del	IFS	de novo	1	D	D	—	Forkhead	0	Pathogenic
FOXP2										
c.620_621insAGC	p.Q207delinsQA	IFS	de novo	1	—	—	—	—	0	Likely pathogenic
c.1217T>C	p.M406T	MIS	inherited	1	D	D	16.75	coiled‐coil	0	VUS
c.1514C>T	p.P505L	MIS	inherited	1	D	D	26.2	Forkhead	1	VUS
c.1607G>C	p.R536P	MIS	de novo	1	D	D	28.5	Forkhead	0	Pathogenic
c.1658G>A	p.R553H	MIS	de novo/inherited	2	D	D	34	Forkhead	0	Pathogenic
c.2084A>C	p.H695P	MIS	de novo/inherited	2	D	T	12.23	—	0	Pathogenic
FOXG1										
c.98A>C	p.H33P	MIS	de novo	1	B	D	21.7	—	1	Likely pathogenic
c.543G>C	p.K181N	MIS	de novo	1	D	D	23.6	Forkhead	0	Pathogenic
c.545C>T	p.P182L	MIS	de novo	1	D	D	33	Forkhead	0	Pathogenic
c.545C>A	p.P182Q	MIS	de novo	1	D	D	31	Forkhead	0	Pathogenic
c.553A>T	p.S185C	MIS	de novo	2	D	D	24.8	Forkhead	0	Pathogenic
c.561C>G	p.N187K	MIS	de novo	1	D	D	26	Forkhead	0	Pathogenic
c.561C>A	p.N187K	MIS	de novo	3	D	D	26	Forkhead	0	Pathogenic
c.565C>T	p.L189F	MIS	de novo	1	D	D	27.2	Forkhead	0	Pathogenic
c.577G>A	p.A193T	MIS	de novo	1	D	D	29.5	Forkhead	0	Pathogenic
c.581T>G	p.I194S	MIS	de novo	1	D	D	29.3	Forkhead	0	Pathogenic
c.590G>T	p.S197I	MIS	de novo	1	D	D	29.4	Forkhead	0	Pathogenic
c.592_594del	p.P198del	IFS	de novo	1	—	—	—	Forkhead	0	Pathogenic
c.643T>C	p.F215L	MIS	de novo	1	D	D	28	Forkhead	0	Pathogenic
c.662A>>G	p.N221S	MIS	de novo	1	D	D		Forkhead	0	Pathogenic
c.673T>C	p.W225R	MIS	de novo	1	D	D	26.7	Forkhead	0	Pathogenic
c.689G>A	p.R230H	MIS	de novo	1	D	D	33	Forkhead	0	Pathogenic
c.700T>C	p.S234P	MIS	de novo	1	D	D	26.3	Forkhead	0	Pathogenic
c.730C>T	p.R244C	MIS	de novo	2	D	D	35	Forkhead	0	Pathogenic
c.755G>A	p.G252D	MIS	de novo	1	D	D	28.4	Forkhead	0	Pathogenic
c.757A>G	p.N253D	MIS	de novo	1	D	D	27.7	Forkhead	0	Pathogenic
c.770T>G	p.L257R	MIS	de novo	1	D	D	27.5	Forkhead	0	Pathogenic
c.821G>A	p.R274Q	MIS	de novo	1	D	D	34	Forkhead	0	Pathogenic
c.1139C>T	p.T380M	MIS	de novo	1	D	D	27.7	—	0	Likely pathogenic
c.1241C>A	p.T414N	MIS	de novo	1	P	T	22.7	—	0	Likely pathogenic

Note

Isoform: *FOXP1*, NM_001244814.1; *FOXP2*, NM_014491.3; *FOXG1*, NM_005249.4. The variant *FOXP1*:p.R514C was reported in two patients with undetermined independence.

Abbreviations: AA, amino acid; gnomAD, Genome Aggregation Database; IFS, inframeshift; MIS, missense; VUS, variants of uncertain significance.

**Figure 3:**
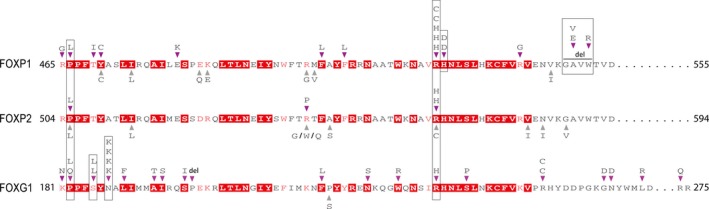
Orthologous peptide sequence alignment of the forkhead domains in *FOXP1* (GenBank accession number: AF146696.1), *FOXP2* (GenBank accession number: KJ903607.1) and *FOXG1* (GenBank accession number: AF146696.1). Variants identified in neurodevelopmental disorder patients are indicated by the inverted triangles above the sequences. Variants identified in Genome Aggregation Database are indicated by the triangle below the sequences. Alternated amino acids were also shown below or above the triangles. Amino acids with red background represent the conserved sites across the three genes

We found that the majority (78%) of the de novo or inherited missense/inframeshift variants was clustered in the forkhead domain (Figure 2) and 36/38 (95%) were classified as pathogenic variants. The amino acid sequences of forkhead domains in *FOXP1* and *FOXP2* are 87% identical. However, the peptide sequences of forkhead in *FOXG1* are significantly different from *FOXP1* (50%) and *FOXP2* (46%) (Figure 3). Importantly, we found that recurrent pathogenic mutations were identified in two sites, which are conserved across all three domains of the three genes. One equivalent site (FOXP1:p.R514, FOXP2:p.R553, FOXG1:p.R230) was identified with mutations in eight independent families (five in FOXP1, two in FOXP2, one in FOXG1). The second equivalent site (FOXP1:p.P466, FOXP2:p.P505, FOXG1:p.P182) was identified with mutations in four independent families (one in FOXP1, one in FOXP2, two in FOXG1) (Figure 3). In addition, two recurrent missense sites, which are conserved only in *FOXP1* and *FOXP2*, were identified in *FOXG1*. One was FOXG1:p.N187K, which was recurrently identified in four independent families, and the other was FOXG1:p.S185L, which was identified in two independent families. This observation indicates that these two sites are particularly important in *FOXG1*. Compared to the sites with mutations identified in NDD patients, the missense variants from gnomAD in the forkhead domains across the three genes are mostly located in the not conserved sites (Figure 3).

In addition to variants in the forkhead domain, there are nine de novo missense variants and one de novo inframeshift variant outside the forkhead domain, including four in *FOXP1*, three in *FOXP2* and three in *FOXG1* (Table 1; Figure 2). All missense/inframeshift variants outside the forkhead domain were classified as likely pathogenic or VUS. Compared to variants in the forkhead domain (all predicted as damaging), approximately one‐third of the variants outside the forkhead domain were predicted as benign/tolerant or probably damaging by SIFT and PolyPhen2. Functional analysis is needed to confirm the pathogenicity of those variants.

## 4 DISCUSSION

FOX genes play important roles in developmental regulation, especially in organogenesis and differentiation of tissues (Kaufmann & Knochel, 1996). The orientation of the dimers requires the protein to bind opposing (nonadjacent) DNA sites, and the FOXP members can form dimers by domain swapping (two monomers interact by exchanging helix H3) (Jackson, Carpenter, Nebert, & Vasiliou, 2010; Stroud et al., 2006). The forkhead domain specifically contains a DBD sequence that can bind to the DNA sequence. DBD sequences have a high degree of homology and can be combined with a specific sequence of DNA elements.

In this study, we identified a de novo missense variant (p.E482K) in FOXP1 in an individual with ID. We found a decrease in mRNA expression by qPCR. We hypothesized that, the missense variant changes the structure of the precursor RNA, affecting the splicing process and leading to a decrease in mRNA levels, that the missense variant affects the stability of the mRNA, leading to mRNA degradation; or that this variant affects the regulation of transcriptional elements, resulting in a decrease in the mRNA. However, there are some limitations of qPCR technology, at least including the following. (a) qPCR has a high sensitivity, so a small error can have a large impact on the final result. When the test group and the control group have a small difference, it is easy to see a false positive (Bustin, 2010). (b) qPCR assumes that the efficiency of individual assays is consistent from one run to another, but in fact, it is difficult to achieve this condition in our experiment (Babu, Kanangat, & Rouse, 1993; Dijkstra, van Kempen, Nagtegaal, & Bustin, 2014). (c) qPCR assumes the effect of any variations on *C_q_* value must be equivalent for reference genes and genes of interest (Dijkstra et al., 2014; Schmittgen & Livak, 2008). (d) When damage is detected, it is not possible to determine the type of DNA lesion present because of the nondiscriminatory nature of qPCR (Hunter, Jung, Di Giulio, & Meyer, 2010; Meyer, 2010). Considering the limitations of qPCR, we performed a western blot and confirmed the decreased expression at the protein level.

Some studies have investigated the functional effect of the de novo missense mutations identified in the NDD‐related FOX genes. Elliot Sollis et al. compared the functional and phenotype outcomes of the same mutation involving the equivalent residue in FOXPs. Functional analysis between FOXP1:p.R514H and FOXP2:p.R553H demonstrated a similar molecular outcome. Aberrant subcellular localization, abnormal transcription factor activity and disruption of protein interactions were observed in both mutations (Sollis et al., 2017). In addition, cellular assays demonstrated that FOXP2:p.R553H results in abnormal localization, loss of DNA binding and transcriptional repression activity. Increased cytoplasmic expression and aggregation have been observed in both FOXP2:p.R553H and FOXP1:p.R514. FOXP1:p.R514H lost the transcriptional repression activity (Sollis et al., 2017; Vernes et al., 2006). Both variants can mislocalize and aggregate wild type FOXP1 and FOXP2 in the nucleus and cytoplasm (Estruch, Graham, Chinnappa, Deriziotis, & Fisher, 2016; Sollis et al., 2016). Another equal position in FOXG1, Arg230His, was reported to affect the affinity of FOXG1 for DNA (Takahashi et al., 2012). In addition, Le Guen et al. (2011) found that FOXG1: p.R244C affects the localization of FOXG1. It is possible that the mislocalization of the pathogenic variants in forkhead domains disrupts the functions of these nuclear domains that participate in the assembly of related splicing factors (Le Guen et al., 2011). These studies suggest that missense variants in the forkhead domain affect the function of the corresponding genes, and the de novo missense/inframeshift variants in forkhead domain are pathogenic in NDDs. However, no study has investigated the potential functional effect of the variants outside the forkhead domain. Functional analysis is needed to determine the pathogenicity of the de novo missense/inframeshift variants outside the forkhead domain in the NDD‐related FOX genes.

In summary, we detected and annotated a novel pathogenic missense variant within the forkhead domain of *FOXP1* detailed the clinical outcome. Importantly, we analyzed the missense/inframeshift variant pattern and the assignment of pathogenicity of the variants for three NDD‐related FOX genes. The pathogenic assignment of the missense and inframeshift variants will be beneficial not only for clinical diagnosis and genetic counseling in clinics but also for the pathogenesis studies when considering developing a personalized treatment strategy.

## CONFLICT OF INTEREST

All of the authors declare no conflict of interest.
